# Optimal Skin-to-Stone Distance Is a Positive Predictor for Successful Outcomes in Upper Ureter Calculi following Extracorporeal Shock Wave Lithotripsy: A Bayesian Model Averaging Approach

**DOI:** 10.1371/journal.pone.0144912

**Published:** 2015-12-14

**Authors:** Kang Su Cho, Hae Do Jung, Won Sik Ham, Doo Yong Chung, Yong Jin Kang, Won Sik Jang, Jong Kyou Kwon, Young Deuk Choi, Joo Yong Lee

**Affiliations:** 1 Department of Urology, Gangnam Severance Hospital, Urological Science Institute, Yonsei University College of Medicine, Seoul, Korea; 2 Department of Urology, Incheon Red Cross Hospital, Incheon, Korea; 3 Department of Urology, Severance Hospital, Urological Science Institute, Yonsei University College of Medicine, Seoul, Korea; 4 Department of Urology, Severance Check-Up, Yonsei University Health System, Seoul, Korea; Sun Yat-sen University, CHINA

## Abstract

**Objectives:**

To investigate whether skin-to-stone distance (SSD), which remains controversial in patients with ureter stones, can be a predicting factor for one session success following extracorporeal shock wave lithotripsy (ESWL) in patients with upper ureter stones.

**Patients and Methods:**

We retrospectively reviewed the medical records of 1,519 patients who underwent their first ESWL between January 2005 and December 2013. Among these patients, 492 had upper ureter stones that measured 4–20 mm and were eligible for our analyses. Maximal stone length, mean stone density (HU), and SSD were determined on pretreatment non-contrast computed tomography (NCCT). For subgroup analyses, patients were divided into four groups. Group 1 consisted of patients with SSD<25^th^ percentile, group 2 consisted of patients with SSD in the 25^th^ to 50^th^ percentile, group 3 patients had SSD in the 50^th^ to 75^th^ percentile, and group 4 patients had SSD≥75^th^ percentile.

**Results:**

In analyses of group 2 patients versus others, there were no statistical differences in mean age, stone length and density. However, the one session success rate in group 2 was higher than other groups (77.9% vs. 67.0%; P = 0.032). The multivariate logistic regression model revealed that shorter stone length, lower stone density, and the group 2 SSD were positive predictors for successful outcomes in ESWL. Using the Bayesian model-averaging approach, longer stone length, lower stone density, and group 2 SSD can be also positive predictors for successful outcomes following ESWL.

**Conclusions:**

Our data indicate that a group 2 SSD of approximately 10 cm is a positive predictor for success following ESWL.

## Introduction

Several parameters can be used to optimize extracorporeal shock wave lithotripsy (ESWL) outcomes, including stone characterization, acoustic coupling, and shock wave rate and sequence [1]. Of these, patient factors, such as stone characterization, can be pretreatment positive predictors for successful ESWL outcomes regardless of procedural factors. In most ESWL cases, stone analyses were not performed; therefore, patient factors should have an important role in pretreatment prediction of stone characteristics and treatment outcomes. In particular, due to the popularity of non-contrast computed tomography (NCCT), patient factors have been accurate predictors in such cases. Several studies have demonstrated that the consistency, size, shape, location, and Hounsfield units (HU) of the ureteral stone, as well as the body mass index (BMI) and skin-to-stone distance (SSD) of patients are significant factors that predict the successful outcome in ESWL [2,3].

Notably, since NCCT replaced intravenous urography as a confirmative tool for urinary stone disease, SSD has been a stronger factor than BMI for predicting the success of ESWL in patients with renal calculi [4]. SSD appears to increase in response to localization of the stone, increased subcutaneous and visceral adipose tissue, and renal parenchyma thickness [5]. However, SSD as predictor of ESWL success remains controversial in patients with ureter stones. Until recently, SSD was a significant factor in half of all published studies. However, in the remaining studies, there was no significant difference in SSD for success or stone-free rate after ESWL [6–11]. Thus, we investigated why SSD was not a predicting factor for successful outcome following ESWL in previous studies. We also determined the optimal SSD, which can be used as a new positive predictor for successful ESWL outcomes in patients with upper ureter stones.

## Materials and Methods

### Patient cohort

Medical records were obtained from a maintained database of patients who had undergone their first session of ESWL between January 2005 and December 2013 at Severance Hospital, Seoul, Korea. During this period, at total of 1,519 patients were registered in our database. Inclusion criteria for the current study were as follows: i) upper ureter stones measuring 4 to 20 mm, ii) radiopaque calculi located within the ureter on simple X-ray within one month of treatment with no evidence of stone migration, and iii) the upper ureter is defined as the segment between the ureteropelvic junction and the superior margin of the sacroiliac joint, and the upper ureter stone is defined as the calculi in the upper ureter [12]. Patients without NCCT scans were excluded. Ultimately, 492 patients with upper ureter stones were eligible for our study.

### Good clinical practice protocols

The study was performed in accordance with applicable laws and regulations, good clinical practices, and the ethical principles described in the Declaration of Helsinki. The Institutional Review Board of Severance Hospital approved this study protocol (Approval No. 4-2015-0398). Written informed consent given by participants was exempted because of the retrospective study design and patients records and information was anonymized and de-identified prior to analysis.

### Extracorporeal shock wave lithotripsy

ESWL was performed using the electroconductive lithotripter (EDAP Sonolith Praktis, Technomed, Lyon, France) until 2011. Since, 2012, it was replaced by the electromagnetic generative lithotriptor (Dornier Compact Delta II lithotripter, Dornier Medtech, Wessling, Germany). All patients were treated under fluoroscopic guidance. The number of shock waves per ESWL session varied from 2500 to 4000 at a rate of 60–90 shock waves per minute. We prematurely terminated the session if the stone became difficult to visualize during the session. The launch intensity was conducted when the focal peak pressure ranged from 16 to 55 MPa as determined by the pain reported by patients while ESWL was being performed.

### Stone characteristics on NCCT

Stone characteristics include location, size, SSD, and mean stone density. The SSD was measured in the axial plane, 45° from the vertical axis. The longest stone length measured on NCCT was used. We used the GE Centricity system (GE Healthcare Bio-Sciences Corp., Piscataway, NJ) during the measurement procedure. The mean stone density was measured using bone windows on the magnified, axial NCCT image of the stone in the maximal diameter, where the elliptical region of interest incorporated the largest cross-sectional area of stone without including adjacent soft tissue. Successful ESWL treatment of ureter and renal calculi was defined as those patients who were rendered stone free or had asymptomatic, clinically insignificant residual fragments ≤3 mm in maximal diameter two weeks after a single ESWL treatment [13] as measured by simple X-ray and did not require auxiliary measures within a 3-month follow-up period.

### Statistical analyses

For subgroup analyses, patients were divided into four groups based on the first, second and third quartiles. Group 1 consisted of patients with SSD <25^th^ percentile, group 2 included patients with SSD in the 25^th^ to 50^th^ percentile, group 3 patients had SSD in the 50^th^ to 75^th^ percentile, and group 4 patients were ≥75^th^ percentile. Statistical comparisons of continuous variables from patient demographic information were performed using Student’s or Welch’s two-sample t-tests. In subgroup analyses, one-way analyses of variance (ANOVA) were used. After ANOVA, Tukey–Kramer’s post hoc tests were used for comparisons between groups. Categorical variables were compared using Pearson's chi-squared tests. Univariate and multivariate logistic regression analyses with a binomial were carried out to define predicting factors following ESWL.

Additionally, we used Bayesian model averaging to identify the best set of predictors for mortality across all feasible models based on Bayesian probability theory. For the multivariable analysis, we compared the commonly used stepwise variable selection approach with Bayesian model averaging, which provided a mechanism for accounting for model uncertainty with the aim of improving prediction accuracy [14,15]. The Bayesian model averaging approach averaged results over multiple models and used the posterior probabilities of these models to perform all inferences and predictions [16]. From these averaged estimates, the posterior probability that a coefficient was nonzero was calculated. The posterior probability, P(B≠0), was interpreted as the probability that a predictor has an effect. The interpretation of P(B≠0) was categorized as follows: <50%, evidence against an effect; 50%–75%, weak evidence of an effect; 75%–95%, positive evidence; 95%–99%, strong evidence; and >99%, very strong evidence [17].

Statistical analyses were performed using R software (version 3.0.3, R Foundation for Statistical Computing, Vienna, Austria; http://www.r-project.org) and its BMA package for the Bayesian model-averaging approach.

## Results

### Demographic analysis in all patients with upper ureter stones and the four groups

The mean age of the 492 patients with upper ureter stones was 51.29±14.32 years. The mean maximal stone length was 9.33±3.85 mm and the mean stone density was 719.70±272.81 HU. The mean HU ratio was 14.20±8.82 HU/mm and the mean SSD was 108.78±19.23. The median SSD (interquartile range) was 108.80 (97.53–121.60) mm. The number of cases with one session success was 343 (69.7%) (Table 1).

**Table 1:** Demographic data on all patients, including patients who were divided into four groups according to SSD percentile.

		SSD groups				
	Total patients	Group 1; < 25^th^ percentile	Group 2; 25^th^ to 50^th^ percentile	Group 3; 50^th^ to 75^th^ percentile	Group 4; ≥75^th^ percentile	P-Value
No. of patients	492	123	122	122	125	
Sex (M:F)	315:177	61:62	68:54	91:31	95:30	<0.001^a^
Mean age (yr)	51.29±14.32	47.28±17.41	52.95±13.26	51.12±12.25	53.78±13.07	0.002^b^
Maximal stone length (mm)	9.33±3.85	9.48±4.02	9.23±3.59	8.65±3.04	9.97±4.50	0.054^b^
Mean stone density (HU)	719.70±272.81	734.68±294.11	751.47±249.65	668.10±268.76	724.30±272.82	0.092^b^
HU ratio (HU/mm)	14.20±8.82	13.96±10.06	14.61±8.51	14.60±7.89	13.66±8.72	0.784^b^
Skin to stone distance (mm)	108.78±19.23	83.35±10.55	103.92±3.50	115.38±3.51	132.10±8.63	-
One session success (%)	343 (69.7)	85 (69.1)	95 (77.9)	85 (69.7)	78 (62.4)	0.071^a^

^a^. Based on Pearson's chi-squared tests with Yates' continuity correction

^b^. Based on one-way ANOVA

**Fig 1:**
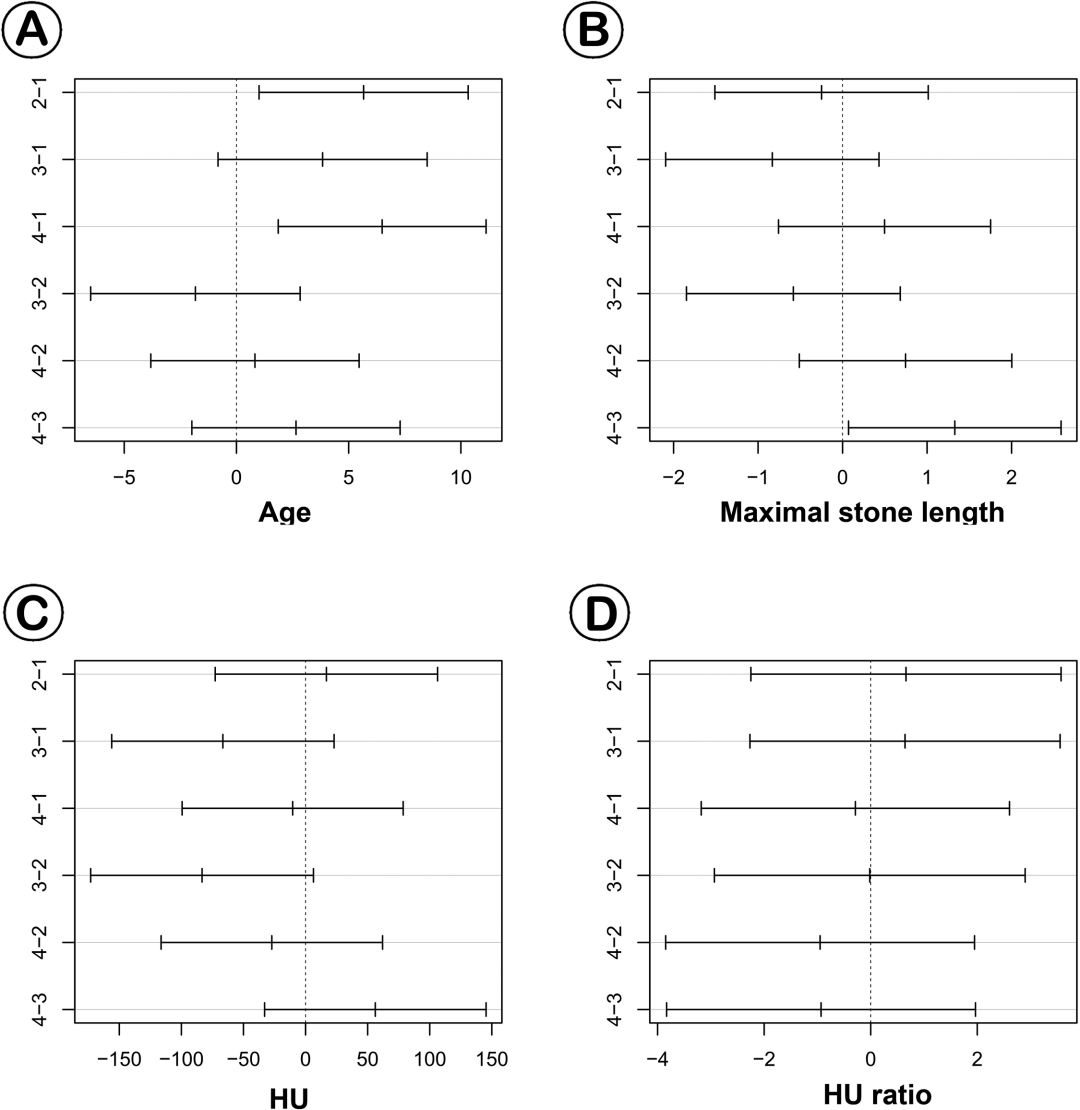
Based on post hoc tests, patients in group 1 were younger than those in groups 2 and 4 (A). Maximal stone lengths were not significantly different; however, post hoc tests showed that the maximal stone length in group 3 was shorter than in group 4 (B). For mean stone density and HU ratio, there were no differences in the four groups (C and D). 1; group 1, 2; group 2, 3; group 3, 4; group 4.

In subgroup analyses, the sex ratio distribution consisted of 61:62 in group 1, 68:54 in group 2, 91:31 in group 3, and 95:30 in group 4 (P<0.001). In groups 1 and 2, percentage of female patients was higher compared to groups 3 and 4. The mean ages in each group were 47.28±17.41 years, 52.95±13.26 years, 51.12±12.25 years, and 53.78±13.07 years, respectively (P = 0.002). Based on post hoc tests, patients in group 1 were younger than those in groups 2 and 4 (Fig 1A). Maximal stone length was not significantly different; however, post hoc tests showed that the maximal stone length in group 3 was shorter than in group 4 (Fig 1B). For mean stone density and HU ratio, there were no differences among the four groups (Fig 1C and 1D). The number of one session success cases in group 2 was higher than other groups. However, there were no significant differences among the groups (Table 1).

### Group 2 versus other groups

The sex ratio distribution in other groups consisted of 247:123, which was significantly different compared to group 2 (P = 0.037). However, there were no statistical differences in mean age, maximal stone length, mean stone density, and HU ratio. The one session success rate in group 2 was higher compared to the other groups (77.9% in group 2 vs. 67.0% in the other groups; P = 0.032) (Table 2).

**Table 2:** Demographic data on all patients, including patients in group 2 and others.

	Group 2; 25^th^ to 50^th^ percentile	Groups 1, 3, and 4	P-value
No. of patients	122	370	
Sex (M:F)	68:54	247:123	0.037^a^
Mean age (yr)	52.95±13.26	50.74±14.63	0.123^b^
Maximal stone length (mm)	9.23±3.59	9.37±3.94	0.711^b^
Mean stone density (HU)	751.47±249.65	709.22±279.55	0.117^b^
HU ratio (HU/mm)	14.61±8.51	14.07±8.92	0.544^b^
SSD (mm)	103.92±3.50	110.38±21.85	-
One session success (%)	95 (77.9)	248 (67.0)	0.032^a^

^a^. Based on Pearson's chi-squared tests with Yates' continuity correction

^b^. Based on student's or Welch's two sample t-tests

**Fig 2:**
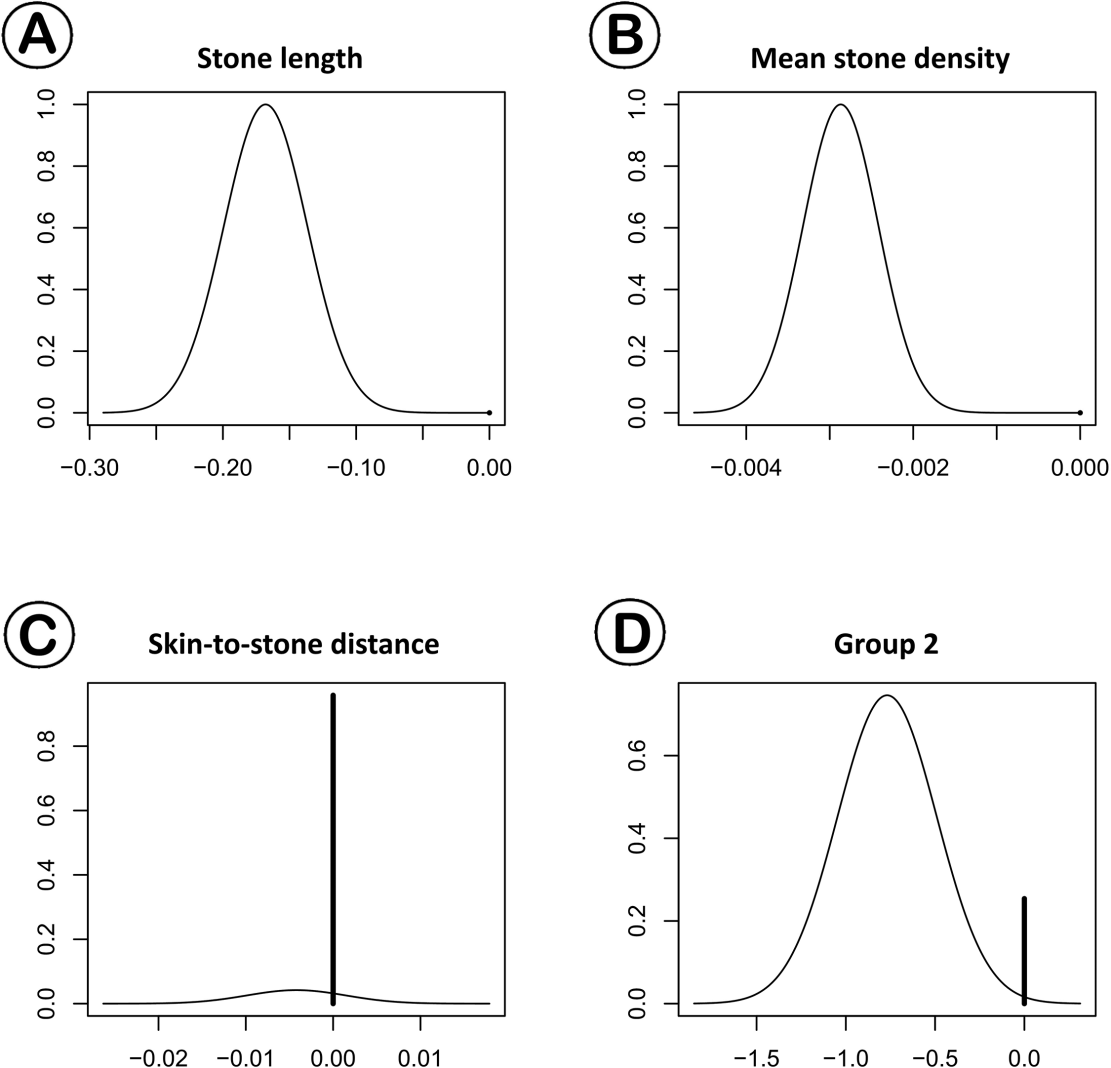
Posterior distribution plots of (A) maximal stone length (MSL), (B) mean stone density, (C) SSD, and (D) group 2 (versus others) in one session success. Shorter maximal stone length, lower mean stone density, and group 2 were also primarily distributed over the zero point for posterior probabilities.

The univariate and multivariate logistic regression models included 492 patients with upper ureter stones, and were performed to examine one session success. Univariate and multivariate models revealed that shorter maximal stone length (OR 0.847, 95% CI: 0.795 to 0.899, P<0.001), lower mean stone density (OR 0.997, 95% CI: 0.996 to 0.998, P<0.001), and group 2 (OR 0.470, 95% CI: 0.267 to 0.803, P = 0.007) were positive predictors for successful outcome in EWSL (Table 3). Using a Bayesian model-averaging approach, shorter maximal stone length [P (B≠0) = 100.0%] and lower mean stone density [P (B≠0) = 100.0%] were the most significant predicting factors for success following ESWL. SSD was not a predicting factor [P (B≠0) = 4.2%]. However, patients in group 2 demonstrated nearly positive evidence for predictor success following ESWL [P (B≠0) = 74.6%] (Table 4). In Fig 2, short maximal stone length, lower mean stone density, and group 2 were primarily distributed over the zero point for posterior probabilities using Bayesian model averaging approach.

**Table 3:** Univariate and multivariate logistic regression model for one session success rate.

	Odds ratio	95% confidential interval	P-value
*Univariate*			
Age	0.992	0.979–1.005	0.259
Male (vs. female)	0.722	0.476–1.085	0.121
Maximal stone length	0.803	0.755–0.851	<0.001
Mean stone density	0.997	0.996–0.997	<0.001
SSD	0.998	0.988–1.008	0.633
Group 2 (vs. others)	0.578	0.353–0.922	0.025
*Multivariate*			
Age	0.997	0.981–1.012	0.692
Male (vs. female)	0.729	0.452–1.164	0.189
Maximal stone length	0.847	0.795–0.899	<0.001
Mean stone density	0.997	0.996–0.998	<0.001
Group 2 (vs. others)	0.470	0.267–0.803	0.007

**Table 4:** Logistic regression model coefficient estimates derived using Bayesian model averaging.

Predictors	Coefficient	P (B≠0)
Maximal stone length	-0.168	100.0
Mean stone density	-0.003	100.0
SSD	-0.001	4.2
Group 2 (vs. others)	-0.573	74.6

P (B≠0): the posterior probability that a coefficient is non-zero

## Discussion

In 2005, Pareek et al. reported that SSD measured by NCCT is a predicting factor for stone-free status of a patient with a lower pole renal stone following ESWL [18]. After distribution of ESWL to treat urinary stone disease, their study was the first to evaluate SSD. Their study measured 64 patients and concluded that SSD is a significant predictor for ESWL outcome (OR 0.32, 95% CI 0.29–0.35; P<0.01).

However, compared with other patient factors, SSD had two basic problems. The first issue regarded race. Because Asian populations have thin body volumes compared to Western populations, it was argued that it could not be applied to Asian patients [19]. Recently, ESWL research conducted in Asia showed that SSD was not a meaningful factor. Ng et al. investigated that the role of NCCT in predicting treatment outcomes of ESWL in patients with upper ureteral stones from Hong Kong [20]. In their demographic data, the mean SSD was 10.23 cm in patients with successful outcomes and 10.44 cm in those with unsuccessful outcomes (P = 0.456). Choi et al. reported predictive factors for failure of ESWL for treating Korean patients with ureteral stones [10]. Using a multivariate logistic regression model, these authors also found that SSD was not a significant predicting factor for failure of ESWL in both groups (stone size ≤10 mm and >10 mm). Tanaka et al. identified the NCCT parameters that best predict the success of ESWL in patients from Japan [11]. They demonstrated that there was no significant difference of SSD between patients with successful and unsuccessful outcomes, and SSD was not a significant predictor for success after ESWL using multivariate logistic regression models. Thus, in Asian populations, SSD may not be a significant predictor for ESWL success or stone-free status.

The second issue was whether SSD could be a predicting factor in ureter stones, which differ from renal stones. Wiesenthal et al. analyzed predicting factors of ESWL success for 422 renal and ureter stone patients and developed a nomogram [21]. They reported that SSD in ureter stones was not an independent predicting factor, and moreover, SSD for ureter stones was longer than for renal stones. Furthermore, the theoretical background that SSD was a predicting factor of ESWL was because as SSD became longer, the shockwave force would be attenuated [22]. Because ureter stones have relatively long SSD compared to renal stones and are located around larger amount of intra-abdominal fat tissue, the prediction power would be decreased [23]. Additionally, in previous studies, one reason that SSD on ESWL successful outcome of ureter stone is a reasonable factor is setting upper ureter stones as well as lower ureter stones as subjects of analyses [8,9,11,24–26]. For lower ureter stones, because SSD should be measured in the anterior abdomen, they had longer SSD than upper ureter stones. Our study analyzed upper ureter calculi because the location of ureter stones could add confusion or bias.

In the current study, patients in group 2 showed statistically better success rates compared to other groups. The reason for this is likely not due to BMI differences according to racial or SSD differences in the calculi. An optimal SSD can be significant, as focusing distance in shock wave energy delivery and stone fragmentation were fixed. In relatively thin patients (e.g. Asian patients), shock wave energy delivery could be interrupted, similar to group 1 (83.35±10.55 mm), which showed the shortest SSD. By focusing the shock waves on a single focal point (F2), the lithotripter concentrates energy at the site where the stone is located [27]. However, a shockwave can easily transfer nonlinear waves [28]. If the SSD is too short for the target stone, it can interrupt focusing through shock wave propagation and induce dispersion. Longitudinal (P) and transverse (S) waves, which have different frequencies and rates, reach the shock wave in F2 repeatedly [29]. Effective stone fragmentation thus begins at the stone surface due to multiple sources of stress.

Given that P and S waves have different frequencies and rates, if the distance to F2 is relatively short, the lithotripter’s focusing of the two waves on F2 could be erroneous. In the electromagnetic shock wave generator, the cylindrical coil surrounded by the cylindrical membrane is designed to focus on F2. However, if the target distance is too short, the shock wave will erroneously focus on the acoustic lens or reflector. This is the primary reason for error in F2. In cases that use an electrohydraulic shock wave generator and are at a short distance from F2, the focus of the ellipsoid reflector on the electrohydraulic lithotripter could be inaccurate. While attempting to maintain the distance to F2 as the water cushion distance expanded, it was difficult to retain patient posture, and focusing became inaccurate. In addition, it decreased the coupling of the shock wave, which could be one reason for the lower success rate. Thus, in contrast to SSD alone, group 2 SSD can be a positive predictor for success following ESWL.

In addition, the SSD in group 2 (mean; 103.92±3.50 mm, range; 97.53–109.70 mm) was consistent with the cutoff values (10–11 cm) used in previous studies [18,24]. However, we proposed that the group 2 SSD of the ureter stone was a predicting factor in ESWL outcome rather than cutoff value. We also confirmed that if the SSD was approximately 10 cm (range; 97.53–109.70 mm), the success rate was higher than other groups. This is likely because as the SSD becomes greater, the shock wave decreases. In relatively thin patients in group 1, the success rate after ESWL may be lower than in patients who have an SSD of approximately 10 cm. Therefore, the SSD for Asian or thin patients in a cohort study could not be considered statistically reasonable.

Our study had some inherent limitations. Its retrospective design may have introduced sampling bias. To overcome its retrospective nature and small sample size, we applied a Bayesian mode-averaging approach, which can reduce bias from standard non-Bayesian approaches. The Bayesian approach is ideally suited for assessing information that accrues during a trial, potentially allowing for smaller yet more informative trials in which patients may receive better treatment [30]. In addition, as a continuous value, SSD was not significant in the logistic regression model, as the optimal distance could be settled. Thus, we suggested that the optimal distance may be the group 2 SSD, which was a significant categorical value in the logistic regression model. The use of two different generating machines may have also resulted in bias; however, there were no statistical difference in successful outcomes for each period. Thus, we suggest that the optimal distance should be approximately 10 cm due to variations in shock wave lithotripters from different companies used at each institute.

Despite these limitations, we are confident in our novel findings regarding the clinical utility of optimal SSD as a predictive factor for successful outcome after patients undergo ESWL for ureteral stones.

## Conclusions

It was previously controversial whether SSD was a predicting factor in the outcome of ESWL in patients with ureteral stones. In our study, we found that group 2 SSD (approximately 10 cm) was a positive predictor for a successful ESWL outcome. A SSD of 8 cm had a decreased one session success rate because once the distance to F2 is too short, the focusing is not optimal.
